# Isolation ssDNA aptamers specific for both live and viable but nonculturable state *Vibrio vulnificus* using whole bacteria-SEILEX technology†

**DOI:** 10.1039/c9ra10733a

**Published:** 2020-04-22

**Authors:** Dejing Liu, Bo Hu, Dingfa Peng, Shan Lu, Shunxiang Gao, Zhengang Li, Lianghua Wang, Binghua Jiao

**Affiliations:** Department of Biochemistry and Molecular Biology, College of Basic Medical Sciences, Second Military Medical University Shanghai 200433 People's Republic of China; Department of Marine Biomedicine and Polar Medicine, Naval Special Medical Center Shanghai People's Republic of China; Eye & ENT Hospital, State Key Laboratory of Medical Neurobiology, Institutes of Brain Science and Collaborative Innovation Center for Brain Science, Shanghai Medical College, Fudan University Shanghai 200032 China

## Abstract

*Vibrio vulnificus* is a ubiquitous marine bacterium that may cause rapid and deadly infection, threatening lives of people living around natural bodies of water, especially in coastal regions. However, traditional culture-based methods are time-consuming and unable to detect Viable But Non-Culturable (VBNC) *V. vulnificus* cells. In this work, we isolated a batch of detection aptamers specifically binding to *V. vulnificus* in all culture status. With traditional whole bacteria-SELEX (Systematic Evolution of Ligands by EXponential enrichment), flow cytometer analysis and imaging, we identify 18 candidates and validated two of them (V8 and V13) as applicable aptamers. Their truncated sequences also showed comparable performance. The dissociation constant (KD) value of V8 is shown to be as low as 11.22 ± 1.32 nM. Optimal aptamers V8 and V13 are also validated to be effective to detect different *Vibrio vulnificus* strains under different binding environments using flow cytometry. As for detection parameters, the LOD of the V8 from cytometry is 29.96 CFU mL^−1^, and the linear range is 10^2^–5 × 10^5^ CFU mL^−1^. This is the first case demonstrating that aptamers can detect the existence of VBNC bacteria as well as live bacteria.

## Abstract 2

With whole-bacteria SELEX, we got aptamers that can bind to *V. vulnificus* in VBNC Status for the first time.

## Graphical abstract

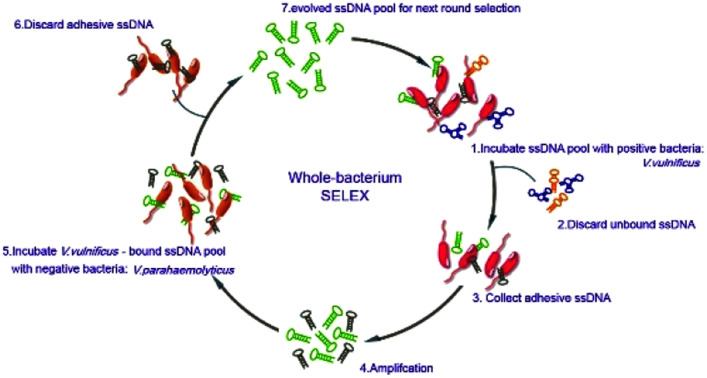

## 1. Introduction

*Vibrio vulnificus* is a Gram-negative, halophilic, flagellated, ubiquitous marine bacterium which can cause serious infection among people all over the world, especially those in coastal states and islands. As part of normal microflora, it can be found in waters, oysters and other shellfish. It is one of the most prevailing marine pathogens. As the FDA (Food and Drug Administration) of the United States reported, there were 459 cases from 1992 to 2007 with a fatality rate of 51.7%.^1,2^ Most cases (85%) occur in the warm water months of May to October in the Northern Hemisphere. People typically get infected through foods. *V. vulnificus* is responsible for 95% of seafood-related deaths in the US. Directly being exposed to contaminated water with a pre-existing lesion or cut can also cause infections. Patients with chronic and underlying diseases, especially liver diseases, are more likely to get infected. Infection symptoms can appear soon after the oyster ingestion, and the onset time can be as soon as 4 h.^3^ If patients can't receive antibiotic treatments in three days, the fatality rate can be 100%.^4^ Thus, monitoring the presence of *V. vulnificus* in waters and seafood is of medical and economic importance.

Current methods for the identification and isolation of *V. vulnificus* from environmental or clinical samples typically rely on selective medium culture, which require further experiments such as PCR to identify the presumptive isolates.^5–7^ Although such methods are quite accurate for the identification and validation of candidate microorganisms like *V. vulnificus*, however, there are still two major shortcomings for using this method in *V. vulnificus* clinical detection:

Firstly, the culture-based methods usually take 3 or 4 days to get the final results, which are quite time-consuming. And considering that patients infected by *V. vulnificus* can only survive three days after infection, it's *IMPOSSIBLE* to use culture-based diagnosis method for such acute urge infection of *V. vulnificus*.

Secondly, under some extreme conditions (like low temperature in winter), *V. vulnificus* has been reported to be able to transformed into a specific state called Viable But Non-Culturable (VBNC) state, making it possible to escape traditional culture based detection. Although with adequate cells (10^8^/mL or more),^8^ the VBNC *V. vulnificus* can be detected through PCR, however, in nature environment, such concentration of VBNC cells can't be reached, and a small number of cells (about 100 cells or less) is enough to cause serious diseases.^9^

Rapid and quick direct detection of pathogens like *V. vulnificus* can be used as a supplement for traditional standard methods to not only provide significant infectious information on patients quickly and effectively, like but also overcome the two shortcomings of traditional methods we mentioned above, making it possible for infected patients to get correct treatment in time.

Multiple subtypes of molecules have been applied for pathogen diagnosis as detectors. Among them, single-stranded nucleic acid can fold into unique and stable structures and makes it an ideal choice. Some single-stranded DNA (ssDNA) or RNA chains can specifically bind to various targets, such as metal ions, small molecules, drugs, proteins, and even whole cells,^10–13^ and they are named as aptamers. Aptamers are very suitable to be developed into diagnostic and therapeutic tools. They can be easily modified with dyes or chemical tools and can be easily immobilized on many kinds of substrates, make it more suitable for quantitative measurements.^14–16^ Compare with antibody, aptamer is smaller, more stable and can tolerate a wide range of temperatures. Additionally, aptamer can be produced *in vitro* without torturing animals and can be rapidly synthesized with high purity and little batch-to-batch variation.

Aptamers are isolated by Systematic Evolution of Ligands by EXponential enrichment (SELEX) procedures.^17^ There are two common strategies to isolate bacteria aptamers, using specific cell surface molecules as targets, or the whole cells as targets. For the first strategy, however, choosing and purifying species-specific membrane molecules can be relatively difficult, and purified molecules may not be able to reserve their native structures when their microenvironments are changed, so their aptamers may not be able to bind to cells. Under such circumstance, the whole-bacteria SELEX approach can be applied and perfectly solves the problem. Whole-bacteria SELEX approach can efficiently screen aptamers with high affinity without tedious molecule purification process, and counter SELEX can ensure the high specificity of the aptamers. In recent years, whole-bacteria SELEX approach has been applied to isolating various bacteria aptamers such as *Mycobacteria tuberculosis*,^18^*Staphylococcus aureus*,^19^*Candida albicans*,^20^*Vibrio. Alginolyticus*,^21^*Vibrio. Parahaemolyticus*,^22^*etc.*

In this study, we employed whole-bacteria SELEX approaches to screen potential ssDNA sequences that can specifically bind to *V. vulnificus*. Based on FACs (Flow Cytometry) and confocal microscopy results, we identified 2 aptamers with good performance, V8 and V13. They can bind to *V. vulnificus* with high binding affinity and can specifically pick *V. vulnificus* out of other bacteria. Such optimal aptamers can detect different *V. vulnificus* strains, tolerate different binding environments, and detect *V. vulnificus* in VBNC status as well as cells in other culture phases. As for the general performance of such aptamer-based detection method, the LOD is 29.96 CFU mL^−1^ and its linear range is 10^2^–5 × 10^5^ CFU mL^−1^. The total test time of this fast screen method is less than 1 hour. Based on our screening methods and analysis results we described above, there are three major innovations that can be summarized in this research field.

Firstly, we developed ssDNA aptamers for *V. vulnificus* with the best binding affinity up to now. The *K*_D_ value for Aptamer V8 is 1.22 ± 1.32 nM, while the previous studie got an aptamer with *K*_D_ = 26.8 ± 5.3 nM;^23^

Secondly, for the first time, we provided an optional method to detect environmental at *V. vulnificus* VBNC status, fulfilling the gaps in this research field.

Thirdly, previous studies generally applied bacteria in one fixed growth period/stage, and they didn't test whether these aptamers can bind to target bacteria in different phases. Therefore, the screened-out aptamers may not be able to identify bacteria if such bacteria entered a different stage, inducing possible false negative results. As we have mentioned above, *V. vulnificus* have various stages. Therefore, it's quite necessary for researcher to screen and validate the candidate aptamers using bacterium from different various stages. However, previous studies on *V. vulnificus* do not contain related screening and analysis. Our study filled the gap and identified the effective aptamers that can be applied to *V. vulnificus* at different phases.

All in all, taking advantages of whole-bacteria SELEX approaches and flow cytometry, in this study, we not only screened out two effective aptamers (V8 and V13) for further clinical and environmental detection on *V. vulnificus* with higher detection speed and accuracy, but also validated their ability to bind to *V. vulnificus* in different stages, especially in VBNC status.

## 2. Results

### Selection of aptamers against *V. vulnificus*

**Fig. 1:**
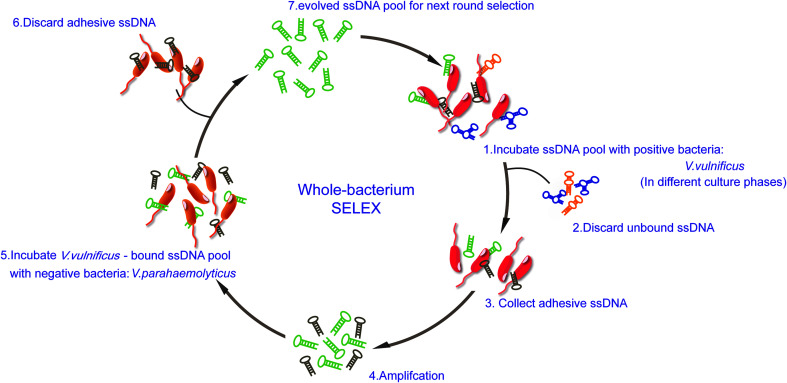
Scheme of *V. vulnificus* aptamer SELEX.

The scheme of whole-bacteria SELEX process was illustrated in Fig. 1. Thirteen rounds of selection were performed to isolate aptamers that can specifically recognize *V. vulnificus*. During the first seven rounds of selection, sequences bound to *V. vulnificus* were collected and amplified. The recovery rates increased gradually, and we decided to introduce counter-SELEX in Round 8 (see ESI Fig. S1†). Through the subsequent six rounds of counter-selection, the unbound sequences and sequences bound to *V. parahaemolyticus* were discarded. The recovery rate of Round 11 increased significantly, indicating more enriched sequences in the system can bind with *V. vulnificus*. 2 more rounds were added for further eliminating non-specific sequences and enrich candidate ones. PCR negative controls were set in each round to avoid possible template contamination, no detectable products yielded. When the whole SELEX process ended, the pool was purified, cloned and sequenced. 80 candidate oligonucleotides were sequenced and their homology and similarity were analyzed *via* Clustal X 2.0. 18 candidate aptamers for further identification (see ESI Table 1†).

### Binding ability analysis of candidate aptamers

**Fig. 2:**
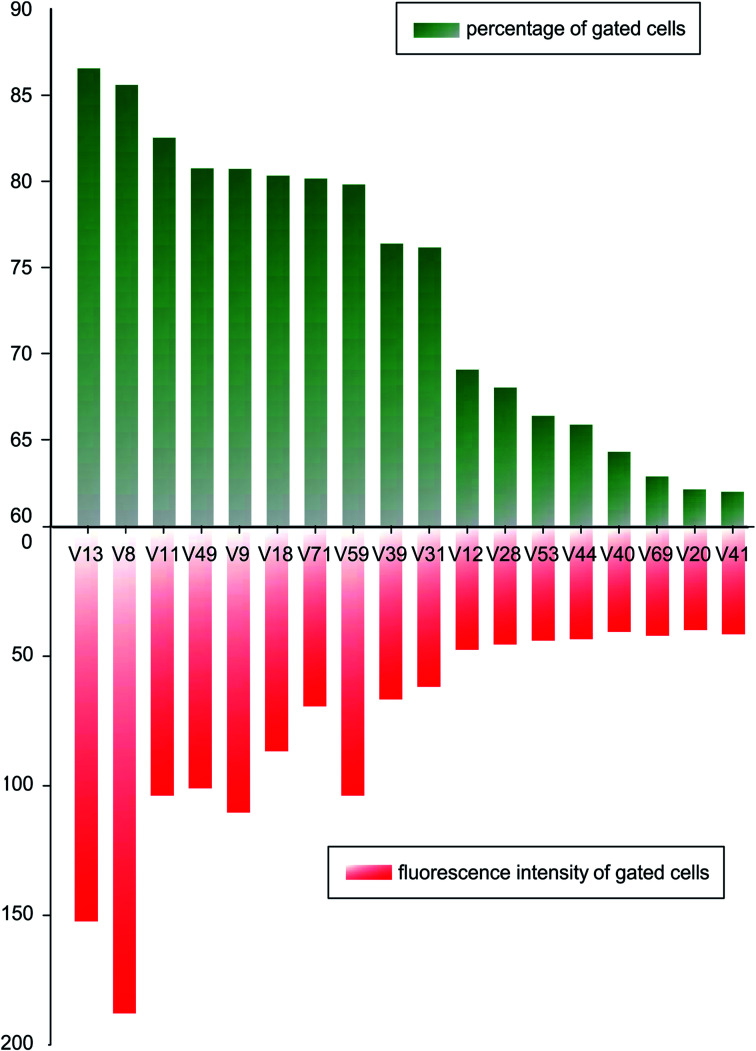
Percentage of fluorescent cells and the mean fluorescent intensity of candidate aptamers bound to *V. vulnificus*.

In order to compare binding ability of the candidate sequences, we carried out flow cytometry to analysis the incubated *V. vulnificus* cells. The candidate aptamers were synthesized with fluorescent labels, when aptamers bind on cells, flow cytometer can detect the fluorescent signals and count the fluorescent cells. Mean fluorescent intensity was used to screen oligonucleotides with higher binding ability. A fluorescently labelled, randomized ssDNA pool was used as background of nonspecific binding. A threshold was set so that the fluorescence intensity of the gated cells would be greater than those incubated with randomized ssDNA pool. 18 tested sequences were incubated with 4 × 10^8^ of *V. vulnificus* cells in binding buffer for 30 min, and the percentage of fluorescent cells and their mean fluorescence intensity are analysed and shown in Fig. 2.

After comparing the gated cell proportions and the mean fluorescence intensity values of the gated cells, we choose V8 and V13 for further study.

### Binding specificity analysis of V8 and V13

**Fig. 3:**
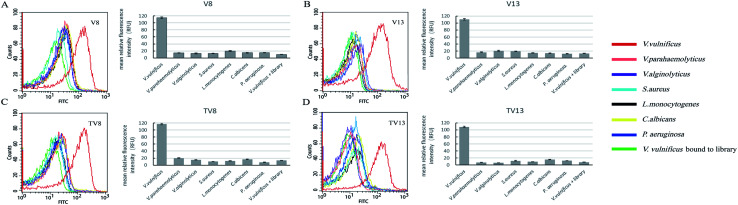
Specificity analysis for *V. vulnificus* aptamers. (A)V8, (B)V13, (C)TV8, (D)TV13 preferentially bind to *V. vulnificus* over other species of bacteria. Bar plot and flow cytometry results are showed simultaneously.

Fluorescently labelled aptamer sequences, V8 and V13, and their truncated sequences, TV8 and TV13 (sequences without primer regions) were incubated with various species of bacteria, including *V. vulnificus*, *V. parahaemolyticus*, *V. alginolyticus*, *S. aureus*, *L. monocytogenes*, *C. albicans*, and *P. aeruginosa*. Fluorescent intensity of different species of bacteria was tested. Fig. 3 clearly shows that when bound to V8 and V13, fluorescent intensity of *V. vulnificus* was significantly greater than other species. Truncated sequences, TV8 and TV13 also inherited the good specificity from V8 and V13, respectively. These results indicated that these sequences can distinguish *V. vulnificus* from other *Vibrio* bacteria as well as other species.

### Fluorescence imaging of *V. vulnificus*-aptamer complex

**Fig. 4:**
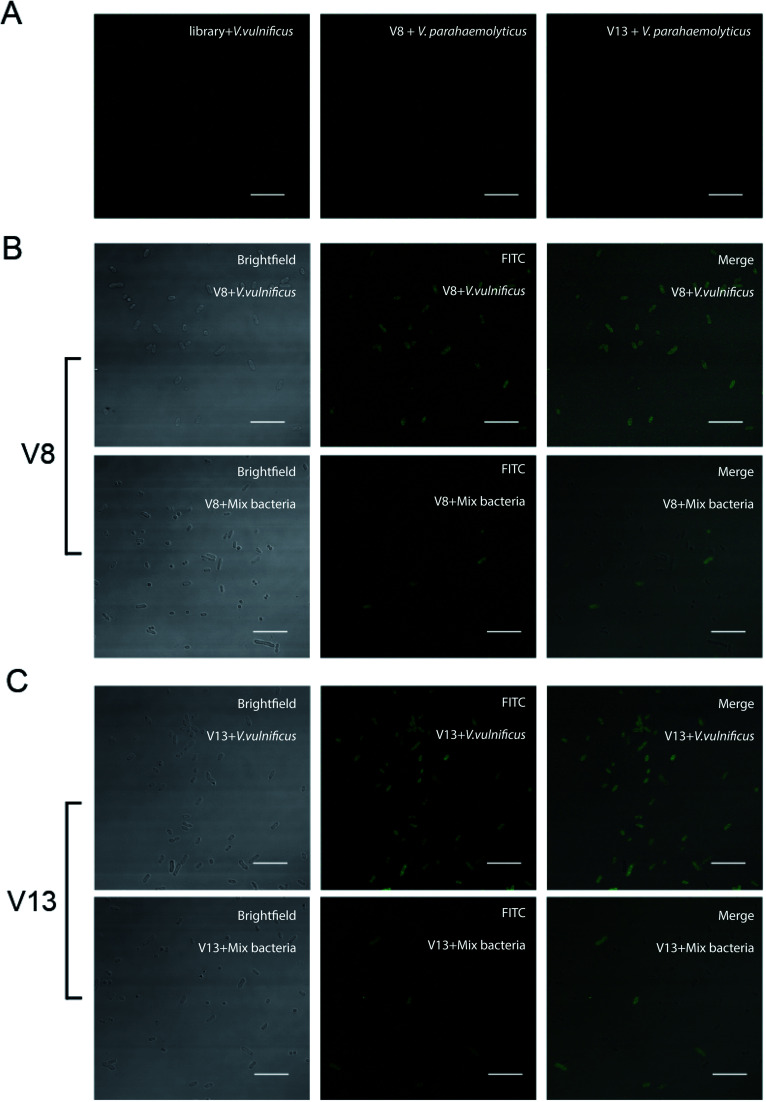
Binding ability of V8 and V13 were evaluated by confocal fluorescence microscopic. (A) Left column, library bind to *V. vulnificus*. Middle column, V8 bind to. *V. parahaemolyticus.* Right column, V13 bind to. *V. parahaemolyticus*. (B) First row, V8 bind to *V. vulnificus*. Second row, V8 incubated with *V. vulnificus*, *S. aureus* and *C. albicans*. (C). First row, V13 bind to *V. vulnificus*. Second row, V13 incubated with *V. vulnificus*, *S. aureus and C. albicans* (scale bar, 10 μm).

In order to further test the binding ability between *V. vulnificus* cells and aptamer, laser scanning confocal microscopy was applied to observe the *V. vulnificus*-aptamer complex. *V. vulnificus* cells were incubated with 4 aptamer sequences respectively. Then the suspension was dropped on glass slide and thin smear was made. Through laser scanning confocal microscopy, we can see clearly that these sequences did binding to the cells efficiently (see Fig. 4 and ESI Fig. S2† online). Little aptamer molecules bound on *V. parahaemolyticus* and *V. alginolyticus*. These results were consistent with the previous flow cytometer results.

### Binding affinity analysis of V8 and V13

**Fig. 5:**
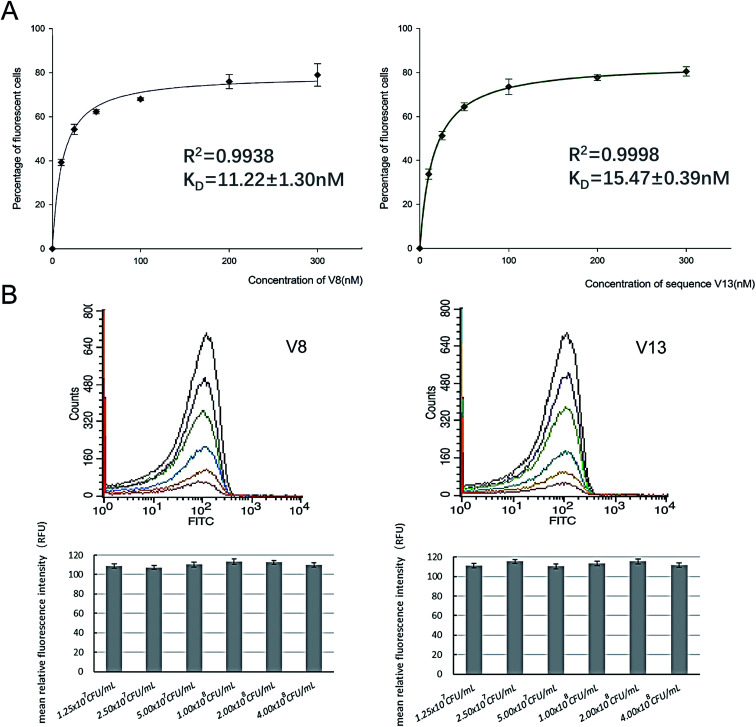
Different concentrations of V8 and V13 bind to a fixed number of *V. vulnificus*, and different concentrations of *V. vulnificus* bind to a certain amount of V8 and V13. (A) Affinities were determined by incubating a fixed, excess number of *V. vulnificus* with a series of different concentrations of V8 and V13. (B) A series of different concentrations of *V. vulnificus* bind to 300 nM V8 and V13. Bar plot and flow cytometry results are showed simultaneously.

**Fig. 6:**
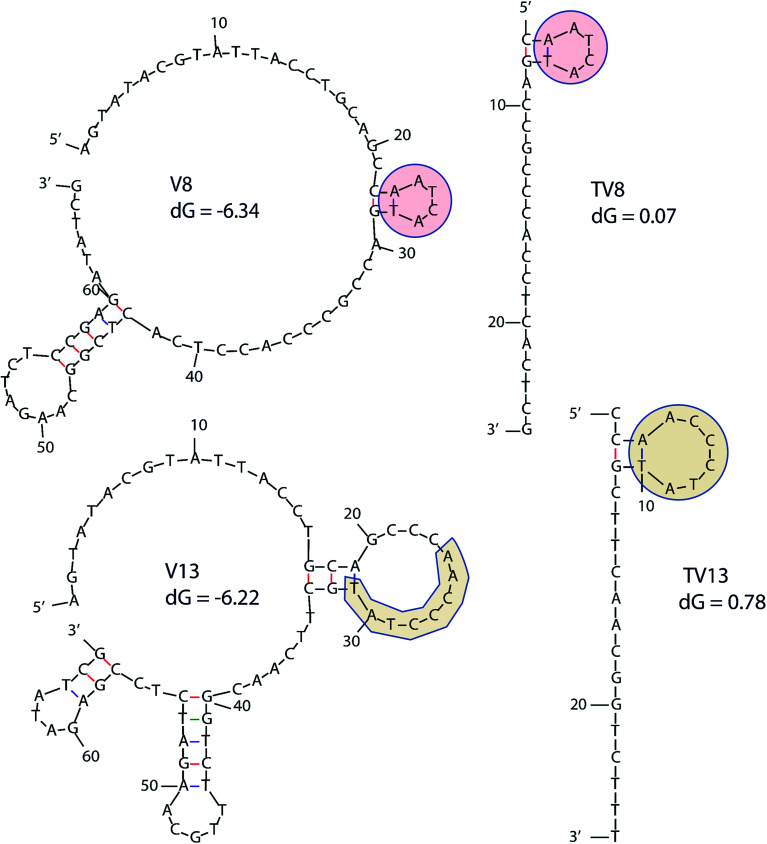
Secondary structure of 4 aptamers. Similar parts are highlighted in the figure. d*G* means initial free energy.

In order to determine equilibrium dissociation constant of V8 and V13, and their truncated sequence TV8 and TV13, *V. vulnificus* cells were incubated with different concentrations of aptamers. Fig. 5(A) shows one site saturation curves based on the flow cytometric analysis results. As Table 1 shows, *K*_D_ values of four sequences reached nanomolar level, and both selected sequences and truncated sequences showed excellent binding capacity. The secondary structure of these 4 aptamers are shown in Fig. 6. V8 and TV8 have the same loop, and part of V13 and TV13 loops are the same. These results suggest that the 25-nt variable regions of the aptamer sequences are more likely to be responsible for binding to the target.

**Table 1:** Summary of the estimated *K*_D_ values of the candidate aptamers. Underline indicates prime regions

Aptamer	Sequence	*K*_*D*_ (nM)
V8	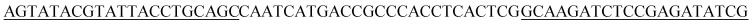	11.22 ± 1.30
TV8	CAATCATGACCGCCCACCTCACTCG	17.44 ± 1.30
V13	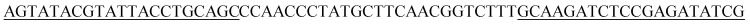	15.47 ± 0.39
TV13	CCAACCCTATGCTTCAACGGTCTTT	13.21 ± 2.19

### V8 and V13 bind to different concentrations of *V. vulnificus*

In order to further investigate binding ability of aptamers against *V. vulnificus*, we incubated 300 nM V8 and V13 with a series of different concentrations of *V. vulnificus*. According to binding affinity analysis results, 300 nM V8 and V13 can achieve binding saturation. As Fig. 5(B) shows, different concentrations of *V. vulnificus* showed similar fluorescent signals when incubating with an excess concentration of aptamers.

### V8 and V13 bind to *V. vulnificus* in all culture status

**Fig. 7:**
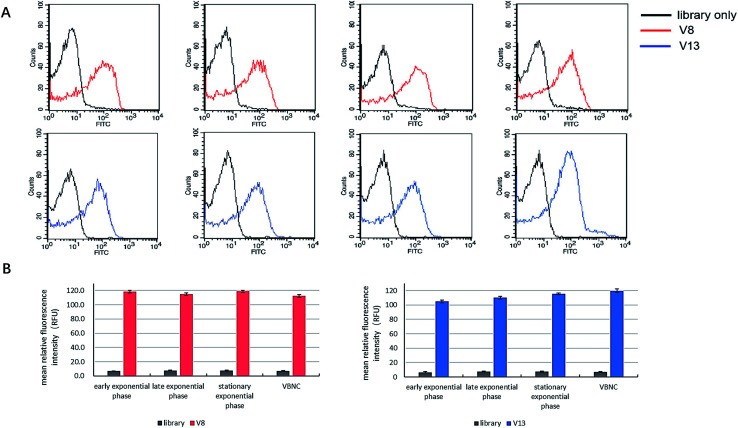
The comparison of binding performance of V8 and V13 on *V. vulnificus* cells in different culture phases. Bar plot and flow cytometry results are showed simultaneously.

Some articles state that aptamers have the potential to be employed to detect bacteria in VBNC status. However, none of them demonstrated this point clearly and directly.^20,24,25^ We tested whether V8 and V13 can bind to *V. vulnificus* in VBNC status as well as other culture status. As Fig. 7 shows, V8 and V13 showed similar performance when bound to *V. vulnificus* in different culture phases.

### V8 and V13 bind to *V. vulnificus* isolated from different sources

Qualified *V. vulnificus* aptamer should have ability to identify all strains of *V. vulnificus,* no matter where they come from. Thus, we tested whether V8 and V13 can also identify *V. vulnificus* MCCC 1A08743 and MCCC 1H00047. MCCC 1A08743 was isolated from a Chinese patient, while MCCC 1H00047 was isolated from estuarine water in China. V8 and V13 can identify two stains of *V. vulnificus* and the signals are quite similar with ATCC27562 (see ESI Fig. S3†(A) online).

### V8 and V13 bind to *V. vulnificus* in different binding environment

We tested whether V8 and V13 can act in complex binding environment as well as in binding buffer. Serum was tested for potential clinical use, and oyster infusion was test for food safety application. V8 and V13 can also recognize *V. vulnificus* in human serum and oyster infusion (see ESI Fig. S3†(B) online). To better validate stability, PAGE gel electrophoresis was also conducted (see ESI Fig. S3(C)† online). No tails below 60 nt was observed. V8 and V13 can tolerate diluted serum as well as oyster infusion.

### The Limit of Blank and Limit of Detection for V8-flow cytometer detection method

The Limit of Blank (LOB)and Limit of Detection (LOD)for V8-flow cytometer detection method is measured and calculated according to CLSI-17A. The LOB is 7 CFU mL^−1^, and the LOD is 29.96 CFU mL^−1^. See the ESI Table 3 and Table 4† for detailed data.

### The linear range of V8-flow cytometer detection method

**Fig. 8:**
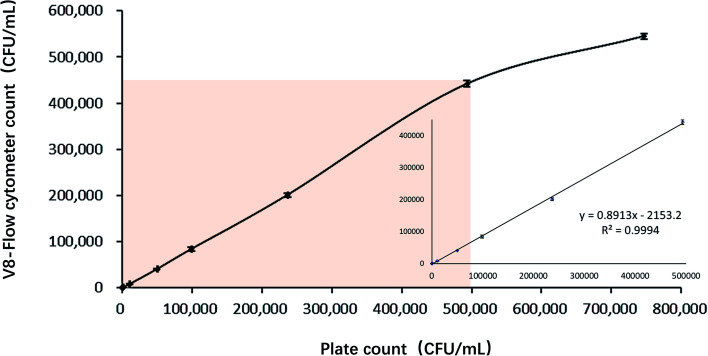
The relationship between V8-flow cytometer detection method and plate counting method in detecting *V. vulnificus* samples.

The linear range of V8-flow cytometer detection method is measured according to CLSI-EP6A.^26^ Good linear relationship between plate count result and V8-flow cytometer detection result at the range between 10^2^ to 5 × 10^5^ CFU mL^−1^ is shown on Fig. 8. The regression coefficient at this range is 0.9994. Flow cytometer loses its resolution at higher concentration, probably because it cannot analyse large number of particles at fast fluid speed.

### Aptamer V8 bind to protein-free *V. vulnificus*

V8 showed best binding affinity and its target molecule was explored. We treated *V. vulnificus* with Proteinase K and trypsin to see whether the target of V8 is protein. If the fluorescence signal reduces, the target of the aptamer may be protein molecules on bacterial cell wall. However, as ESI Fig. S4† online shows, the treatments did not cause significant signal change. This result suggested that the target might not be membrane protein, but other cell wall composition, such as LPS, capsule polysaccharide or other kinds of molecules.

## 3. Discussion

All in all, as we have described above, we applied modified whole-bacteria SELEX and got aptamers that can specifically bind to *V. vulnificus*. The accuracy and efficacy of the optimal aptamers have also been validated under different circumstance and against different strains of *V. vulnificus*, and the aptamers showed excellent performance. Here, in this study, we divided further discussion on our project into three parts: methodological innovations, clinical significance of new aptamers and further perspectives in this field.

For methodological innovations, we have made three major innovations during the selection, validation and application of the optimal aptamers against *V. vulnificus*.

First, in this study, we emphasized on developing aptamers that can bind to different stages of *V. vulnificus*. In previous studies, lots of aptamers against bacteria were selected by SELEX and many of them were developed into splendid detection method.^24,27–30^ However, most of them failed to pay attention to bacteria at different stages and relied on single phase of bacteria for aptamer selection and validation. Then, some scholar started to noticed the phase differences. Ying Zou *et al.* developed aptamers that can bind to different stages of *E. coli* O157:H7.^31^ Soo HwanSuh *et al.* developed aptamers specifically targeted bacterial cells at different growth phases.^32^ Thus, we innovatively used *V. vulnificus* at different stages for positive SELEX, and used different stages of bacteria to validate the aptamers. We especially validated aptamers' binding ability to VBNC state. We identified aptamers with high affinity against different phases of bacteria, improving the accuracy and efficacy for aptamer selection.

Moreover, we chose *V. parahaemolyticus* as counter selection targets to confer the optimal aptamers potential clinical significance. Previously, the aptamer selection for *V. vulnificus* has been reported by Yan, *et al.*^33^ Their work was more focused on fish diseases and they used *V. anguillarum* for counter-SELEX, for further better distinguishing different water pollution pathogens for fish. While in this study, we chose *V. parahaemolyticus* with more clinical significance for the counter selection, making the optimal aptamers effective enough to be applied in the differential diagnosis between *V. vulnificus*. and *V. parahaemolyticus*. For the first time, we identified effective aptamers for *V. vulnificus* with potential clinical significance.

Third, we developed an effective, fast and labour-saving method to detect *V. vulnificus* in VBNC status. In this work we used flow cytometer as a part of detection system. Compare to other existing proved VBNC detection methods, such as PCR,^34^ qPCR,^35^ PMA-LAMP (propidium monoazide- loop-mediated isothermal amplification)^36,37^ PMA-based qPCR,^38^ CRENAME + rtPCR^39^ (rapid concentration and recovery of microbial particles, extraction of nucleic acids and molecular enrichment), the LOD of flow cytometer is slightly higher or equivalent. There is a trade-off between “easy to operate “and” extreme sensitivity”. LOD of CRENAME + rtPCR can reach to 1.2 CFU/100 mL, but this approach takes 6 h to get the results. V8-flow cytometer detection is fast, with <1 h total test time and easier sample preparation.

For biological and clinical significance of new aptamers, as we have already analysed, the optimal aptamers we screened can be further applied and modified for clinical use. Therefore, according to related publications, we compared the performance of our optimal aptamers with previous proved detection methods. In 2018, Yan, *et al.*^33^ presented an effective detection method and identified effective aptamers which we have already mentioned above. Comparing to this study, we have two advantages:

Firstly, comparing to their optimal aptamer Vapt2 with binding affinity at 26.8 ± 5.3 nM, our optimal aptamer (V8) has a better and more stable performance with binding affinity as 11.22 ± 1.30 nM. Secondly, different from previous studies using microscope to detect, which is labour-consuming and less quantitative in practical microbe detection, we applied flow cytometry to detect the performance of aptamers, which is quite fast, convenient for sample preparation and more suitable for further clinical applications.

As for future perspectives, we may still focus on the optimal aptamers we screened out in this study like aptamer V8 and V13. There are two major directions that we may contribute to in the future:

Firstly, we will try to reveal the detailed biological mechanism for the aptamer to bind the target bacteria. In this study, we have already applied related experiments to make the preliminary trials in this field. Using optimal aptamer V8 and cultured *V. vulnificus* strains, we incubated V8 with protein-free cells. And after incubation fluorescent did not change much, indicating that the target molecule of aptamer V8 is not protein but another cell wall component. Since the expression of some outer membrane protein may alter in different culture phases, it is reasonable that our SELEX strategy yielded aptamers that can bind to component that expresses on most *V. vulnificus* cells. Further studies are needed to identify the exact target molecule of V8.

Secondly, we will try to develop a reliable and fast detection kit using the aptamers for the detection of *V. vulnificus* at different stages. Although based on our experiments, V8-cytometry method have already been validated to be effective and accurate enough for the identification of *V. vulnificus* at different stages, more modification should be made and a more efficient and sensitive biosensor or clinical diagnosis kit should be established. More experiments should be applied to make sure the stability and accuracy of our kit in clinics or for environment monitoring. Therefore, we may focus on the development of novel *V. vulnificus* detection methods, by applying technologies like aptamer-conjugated nanoparticles, quantum dots or colorimetric approaches to increase the sensitivity, handleability and throughput of the detections.

## 4. Materials and methods

### Bacterial strains and culture media

*V. vulnificus* ATCC 27562, *V. parahaemolyticus* ATCC17802 and *V. alginolyticus* ATCC 17749 were obtained from American Type Culture Collection (ATCC), Georgetown, DC, USA. *V. vulnificus* MCCC 1A08743 and *V. vulnificus* MCCC 1H00047 were obtained from Marine Culture Collection of China. *Vibrio* bacteria were grown in brain heart infusion medium (Land Bridge, Beijing) with 3% NaCl at 37 °C. *V. vulnificus* in three different culture phases early exponential phase (OD_600_ = 0.3), late exponential phase (OD_600_ = 0.7), and stationary phase (OD_600_ > 1) were mixed at the ratio of 1 : 1 : 1 in number. The mixture was used for positive SELEX. *V. parahaemolyticus* was cultured and prepared the same as *V. vulnificus* for counter-SELEX.

The following bacteria were also used to identify the specificity of the isolated aptamers: *Staphylococcus aureus* ATCC25923, *Listeria monocytogenes* ATCC19115, *Candida albicans* ATCC10231, and *Pseudomonas aeruginosa* ATCC27853. All these bacteria were cultured overnight under aerobic condition in brain heart infusion media (Land Bridge, Beijing) with 1% NaCl at 37 °C and 150 rpm shaking. All these bacterial strains were obtained from ATCC.

*V. vulnificus* under VBNC status was obtained by refrigerating cultured cells in artificial seawater (ASW) under 4 °C for 7 or more days. The viability was tested on culture plates and growth should not be detected. The VBNC cells were subject to PI stain and showed negative results in flow cytometer.

Membrane protein was removed as previously described with a little modification.^40^ 15 min of trypsin treatment (0.25%, 37 °C) and 10 min proteinase K treatment (1 mg mL^−1^, 65 °C) were conducted. The reaction was stopped by adding Phenylmethanesulfonyl fluoride (PMSF, Beyotime, China) into the suspension.

### Random DNA library and primers

The initial single strand DNA (ssDNA) library and the primers used to amplify DNA were all obtained from Sangon Biotech (Shanghai, China). This ssDNA pool consisted of a central randomized region of 25 nucleotides flanked on both sides by primer regions for amplification,

5′-AGTATACGTATTACCTGCAGC-**N25**-GCAAGATCTCCGAGATATCG-3′ (66-mer).

The ssDNA library contained a maximum of 10^15^ different sequences, which represents high sequence diversity.

Forward primer was 5′-AGTATACGTATTACCTGCAGC-3′.

Reverse primer was 5′-CGATATCTCGGAGATCTTGC-3′.

A poly A-labelled reverse primer (5′-AAAAAAAAAAAAAAAAAAAA-Spacer 18-CGATATCTCGGAGATCTTGC-3′) was used together with unmodified forward primer in PCR to get the asymmetry double strand DNA and to enable further purification of ssDNA by DNA PAGE. Unmodified forward primer and reverse primer were also used for PCR amplification after the final round of the selection for cloning sequencing. GoTaqHot® Start Colorless Master Mix used in PCR was purchased from Promega Corporation. A fluorescent labelled random ssDNA pool was also used as negative binding control.

### Aptamer selection

The whole bacterial cell-SELEX process was performed according to previous reports with a few modifications.^19,21,22^ Briefly, for the SELEX screening, specific concentrations of ssDNA pool dissolved in 100 μL binding buffer (pH 7.4, 0.1 mg mL^−1^ salmon sperm DNA, 1% bovine serum albumin (BSA), 50 mM Tris–HCl, 100 mM NaCl, 1 mM MgCl_2_, 5 mM KCl) were heated to 95 °C for 10 min and cooled in an ice bath for 5 min to form the optimal structural conformation of oligonucleotides. The cell mixture containing 4 × 10^8^*V. vulnificus* cells was incubated with the denatured ssDNA pool at 4 °C for 2 h with end-over-end rotation, allowing the potential aptamer sequences to bind with cells. After the binding reaction, unbound ssDNA was removed by washing three times in 1 mL of binding buffer by centrifugation at 12 000 rpm for 3 min. The amounts of unbound ssDNA were measured and recovery rates were calculated. The cell pellets with adhesive ssDNA were resuspended in water, and PCR reagents were added directly to the cell suspension to amplify the tightly bound DNA sequences on the cells (more details can be found online in ESI Table 2†).

From 8^th^ selection round, the counter-SELEX was incorporated with the positive-SELEX to eliminate false-positive binding of sequences. In the counter-SELEX process, 2 × 10^9^*V. parahaemolyticus* cells were initially incubated with 200 pmol of the denatured ssDNA library at 4 °C. After centrifugation, the unbound oligonucleotides in supernatant were collected and subsequently incubated with *V. vulnificus* at 4 °C. Then unbound ssDNA was removed by centrifugation, adhesive cells were resuspended, and ssDNA was amplified by PCR.

PCR products were further separated into ssDNA chains by 12% urea denaturing PAGE, and ssDNA chains were recovered from the gel band. The eluted ssDNA in binding buffer was collected and purified by Gel extraction kit (QIAEN, Germany). Finally, the purified ssDNA was quantified using a Qubit® 2.0 Fluorometer and used for the next selection round.

### Cloning and sequencing of selected DNA

When the 13^th^ round ended, ssDNA sequences selected by whole-bacteria SELEX were amplified with unmodified primers through PCR. The purified PCR products were then cloned and sequenced by Shanghai Sangon Biological Science and Technology Company (Shanghai, China). These sequences were aligned and analyzed using the Clustal X 2.0 software.

### Flow cytometry analysis

BD FACSCalibur flow cytometer and CellQuest pro software (Becton, Dickinson and Company, American) were used to assess the binding performance of different sequences. Fluorescence intensity of incubated bacterial cells were measured *via* flow cytometry.

Candidate aptamer sequences were labelled with the fluorophore (5′-FAM). A randomized ssDNA pool with fluorescent label was used as a control for nonspecific binding in each experiment. A threshold based on fluorescence intensity was set so that the fluorescence intensity of the gated cells would be greater than those incubated with randomized ssDNA pool. Gated cells were counted and gated fluorescence intensity was quantified. Candidate sequences were dissolved in binding buffer (pH 7.4, 50 mM Tris–HCl, 100 mM NaCl, 1 mM MgCl_2_, 5 mM KCl), preheated, and then incubated with bacterial cells. After incubating with 300 nM aptamer/random ssDNA pool for 30 min, the cells were washed once and resuspended in 300 μL binding buffer for immediate flow cytometric analysis.

Binding curves were created to estimate dissociation constants (*K*_D_) values by incubating different concentrations of aptamers (0–300 nM) with a fixed number of cells (10^8^ CFU mL^−1^).The *K*_D_ were calculated with the equation *y* = *B*_max_*x*/(*K*_D_ + *x*), using SigmaPlot 12.5 software.

To evaluate whether different concentrations of *V. vulnificus* can affect the fluorescent signals when bind with V8 and V13, different concentrations of *V. vulnificus* (1.25 × 10^7^, 2.50 × 10^7^, 5.00 × 10^7^, 1.00 × 10^8^, 2.00 × 10^8^, 4.00 × 10^8^ CFU mL^−1^) were incubated with 300 nM V8 and V13 for 30 min at 25 °C. When tested on flow cytometer, set test time as 60 s.

To measure whether V8 and V13 can bind to *V. vulnificus* in all culture status, 300 nM aptamers were incubated with *V. vulnificus* in early exponential phase, late exponential phase, stationary phase and VBNC status (10^8^ CFU mL^−1^) at 25 °C. for 30 min, respectively.

To analyse whether V8 and V13 can bind to *V. vulnificus* isolated from different resources, 300 nM V8 and V13 were incubated with *V. vulnificus* MCCC 1A08743 and MCCC 1H00047 (10^8^ CFU mL^−1^) at 25 °C for 30 min, respectively.

To analyse aptamer stability, bacterial cells (10^8^ CFU mL^−1^) were incubated with 300 nM V8 and V13 in human serum and oyster infusion. To minimize the influence, the sediments in liquids were removed from serum and infusion by centrifugation at 12 000 rpm for 10 min. For serum incubation, serum was diluted to 30% with binding buffer. After incubation, the samples were directly detected by flow cytometer.

To find out target molecular of V8, 10 min-proteinase K-treated *V. vulnificus* and 15 min-trypsin-treated *V. vulnificus* were incubated with 300 nM V8 at 25 °C for 30 min. Intact *V. vulnificus* was used as control. All the cells are at 10^8^ CFU mL^−1^.

The LOB and LOD is measured according to CLSI-17A.^41^ To test the LOB of V8-flow cytometer detection method, 40 blank samples (300 nM V8 solution) were tested. Kolmogorov-Smirnov test shows that *P* = 0.022 < 0.05, indicates the data does not conform to the normal distribution. According to ISO suggestion, the calculation formula should be LOB = the measured value of the [*N*_B_(*p*/100) + 0.5] bit, *α* = 5%, *p* = 95, that is, the measured value ranked 38th. 5 different low concentrations of samples were tested to measure LOD. 300 nM aptamers were incubated with *V. vulnificus* at the concentrations of 25 CFU mL^−1^, 50 CFU mL^−1^, 100 CFU mL^−1^, 150 CFU mL^−1^, 300 CFU mL^−1^, and the volume of each sample is 12 mL. The sample is divided into 12 equal parts and the cell number in 1 mL was measured for 12 times, then the standard deviations (SD) of these tests were calculated. The formula is LoD_tent_ = LoB + *c*_β_ SD_S_, where SD_S_ is the estimated deviation of the population standard deviation (SD_S_^2^ = (*n*_1_SD_s_1__^2^ + *n*_2_SD_s_2__^2^ + *n*_3_SD_s_3__^2^ + ……+ *n*_*n*_SD_s*n*_^2^)/(*n*_1_ + *n*_2_ + *n*_3_ ……. + *n*_*n*_), *n*_*n*_ = *N*_n_ − 1), *c*_β_ = 1.645/(1–1/(4 × *f*)), *f* is the degree of the freedom, *f* = *N*_S_ − *K*. *K* is the number of groups. *f* = 55, *c*_β_ = 1.6525.

The linear range is determined by reference to the CLSI-EP6A document.^26^ 10^6^ CFU mL^−1^ of *Vibrio vulnificus* suspension was incubated with 300 nM V8 and serially diluted into 9 concentrations. Suspension of each concentration was divided into 6 parts, 3 parts were subjected to flow cytometry detection, and 3 parts were subjected to plate colony counting method. Samples of different concentrations of bacterial suspension should be randomly tested and recorded. The mean number of bacteria in each concentration of the bacterial suspension was fitted to the curve, and the corresponding range of the linear segment was selected as the linear range.

### Fluorescence imaging of *V. vulnificus* to fluorescently labelled DNA aptamers

Fluorescently labelled 5′-FAM-ssDNA aptamers (300 nM) were incubated individually with *V. vulnificus* cells in binding buffer for 30 min at 4 °C. The concentration of *V. vulnificus* is 2 × 10^9^ CFU mL^−1^. BSA and salmon sperm DNA were added into the binding buffer to avoid nonspecific binding. Cells were washed twice to remove the unbound aptamers by centrifugation and resuspended in 15 μL binding buffer. Then, the suspension was dropped on poly-l-lysine-coated slides. Poly-l-lysine-coated slides can avoid cell moving during scanning process. Fluorescent images of bacteria with each aptamer were observed under a fluorescence microscope (Olympus, Japan) using excitation at 488 nm, with 60× magnification.

### Secondary structures prediction

Secondary structures were predicted according to M. Zuker ‘s method.^42^ Set DNA sequence to be linear, folding temperature to be 25 °C, and set Ionic conditions [Na^+^] = 100 mM and [Mg^2+^] = 1 mM.

### DNA PAGE

To test the stability, after incubated with diluted serum and oyster infusion for 30 min, 10 μL 300 nM V8 and V13 were subject to 10% urea denaturing PAGE electrophoresis to see if there was any degradation. The electrophoresis time was 30 min. 20bp DNA Lander (Takara) was used for ssDNA length indication.

## 5. Conclusions

Dependent on whole-bacteria SELEX technology, we screened out ssDNA sequences as potential aptamers binding to *V. vulnificus*. Two of them as aptamers with higher sensitivity and specificity were further confirmed to have quite good performance even in truncated forms. What's more, the performance of V8 has been confirmed to not be affected by the different strains and culture environments and *V. vulnificus* even in VBNC status can still be effectively detected, revealing the potentials of them to be developed as biosensors detecting the existence of *V. vulnificus* clinically or environmentally. In the future, we will focus on the development of more efficient and sensitive biosensors or clinical diagnosis kits.

All in all, we developed *V. vulnificus* detection aptamers and identified two effective aptamers (V8 and V13) with both clinical and environmental detection significance, promoting the development of related technologies and researches in the field of bacterial aptamers. It is the first time for aptamer to be demonstrated its ability to detect VBNC bacteria.

## Conflicts of interest

There are no conflicts to declare.

## Supplementary Material

RA-010-C9RA10733A-s001
