# Supplementary material for: Isolation ssDNA aptamers specific for both live and viable but nonculturable state Vibrio vulnificus using whole bacteria-SEILEX technology
Source: RSC Adv. 2020 Apr 22;10(27):15997–6008. doi: 10.1039/c9ra10733a (PMC9052868; doi:10.1039/c9ra10733a)
Supplement: RA-010-C9RA10733A-s001 [file RA-010-C9RA10733A-s001.pdf]

Electronic Supplementary Material (ESI) for RSC Advances.
This journal is © The Royal Society of Chemistry 2020

Isolation ssDNA Aptamers Specific for both Live and Viable but Nonculturable State *Vibrio vulnificus* using Whole Bacteria-SELEX Technology

Dejing Liu, Bo Hu, Dingfa Peng, Shan Lu, Shunxiang Gao, Shengqun Ouyang, Zhengang Li, Lianghua Wang, and Binghua Jiao

No.	Sequences	Repeat
V08	AGTATACGTATTACCTGCAGCCAATCATGACCGCCCACCTCACTCGGCAAGATCTCCGAGATATCG	2
V09	AGTATACGTATTACCTGCAGCCCTGGACATCATTGAGTACTCGTCTGCAAGATCTCCGAGATATCG	1
V11	AGTATACGTATTACCTGCAGCTCCCAACCAATACCAGTACGTTGTAGCAAGATCTCCGAGATATCG	1
V12	AGTATACGTATTACCTGCAGCTATGGATTTGCGTCATGTTTATGTGGCAAGATCTCCGAGATATCG	1
V13	AGTATACGTATTACCTGCAGCCCAACCCTATGCTTCAACGGTCTTTGCAAGATCTCCGAGATATCG	3
V18	AGTATACGTATTACCTGCAGCTGTGGGTGGGTGGGTGGTATCTGCAGCAAGATCTCCGAGATATCG	2
V20	AGTATACGTATTACCTGCAGCCATCCCCTCTCCTGTTGCCCTGACAGCAAGATCTCCGAGATATCG	1
V28	AGTATACGTATTACCTGCAGCCCTGGACATCATTGAGTACTCGTCTGCAAGATCTCCGAGATATCG	2
V31	AGTATACGTATTACCTGCAGCTGTGGGTGGGATTAGGTTCGGGTGGGCAAGATCTCCGAGATATCG	1
V38	AGTATACGTATTACCTGCAGCCCAGACTTCAATCGCGTCAACCGTTGCAAGATCTCCGAGATATCG	1
V39	AGTATACGTATTACCTGCAGCTGATGGTTGTATGACTGGATGTCAAGCAAGATCTCCGAGATATCG	1
V40	AGTATACGTATTACCTGCAGCTCCCCTTTGCATGGCGGTGACACTGGCAAGATCTCCGAGATATCG	2
V41	AGTATACGTATTACCTGCAGCCACCTAGAACACATTGCAACATTAGGCAAGATCTCCGAGATATCG	2
V44	AGTATACGTATTACCTGCAGCTGCTCCTCGACTGTTGTTAATCGTGGCAAGATCTCCGAGATATCG	1
V49	AGTATACGTATTACCTGCAGCTGACATCGTCTGACCTCCACAAGCAGCAAGATCTCCGAGATATCG	1
V53	AGTATACGTATTACCTGCAGCTGGGTCCGTATGTTGGTGTATGTGAGCAAGATCTCCGAGATATCG	1
V59	AGTATACGTATTACCTGCAGCTGTATACCCGACCGTACCGACGTAAGCAAGATCTCCGAGATATCG	1
V69	AGTATACGTATTACCTGCAGCTCACCTTCACACACTCCCTTCTTCGGCAAGATCTCCGAGATATCG	2
V71	AGTATACGTATTACCTGCAGCCCTGTACAAGCAGTATGTCAGCTGAGCAAGATCTCCGAGATATCG	1

Supplementary Table 1. Candidate aptamer sequences

Selection round	Amount of ssDNA pool (pmol)	Negative selections	Counter-SELEX incubation time(min)	Positive SELEX incubation time (min)	Wash times after incubation(min)
1-7	2000	×	/	120	3
8-10	200	√	120	120	4
10-13	150	√	180	60	5

Supplementary Table 2. Summary of selection protocol for *V. vulnificus* whole-bacteria SELEX

Values of 40 blank samples detections							
0	0	0	0	0	0	0	0
0	0	0	0	0	0	0	0
0	0	1	1	1	1	1	1
2	2	2	2	3	3	3	4
4	4	5	5	6	7	7	7

Supplementary Table 3. Values of 40 blank samples detected by V8-flow cytometer method, sorted in ascending order, from left to right, top to bottom. The 38th value is the LOB.

Conc.	Values of 12 repeat detections												Mean	SD
**25cfu/ml**	27	24	56	37	46	25	46	35	59	47	28	36	38	38.77	11.42
**50cfu/ml**	44	73	60	37	57	70	40	66	59	58	59	69	57	57.61	11.26
**100cfu/ml**	87	89	112	122	95	92	81	115	108	124	107	89	102	101.77	14.04
**150cfu/ml**	163	185	138	136	145	138	165	153	143	175	152	168	158	155.31	15.38
**300cfu/ml**	285	297	278	303	296	309	316	312	329	311	322	303	302	304.85	14.08

Supplementary Table 4. Low concentrations of measurement for LOD calculation.

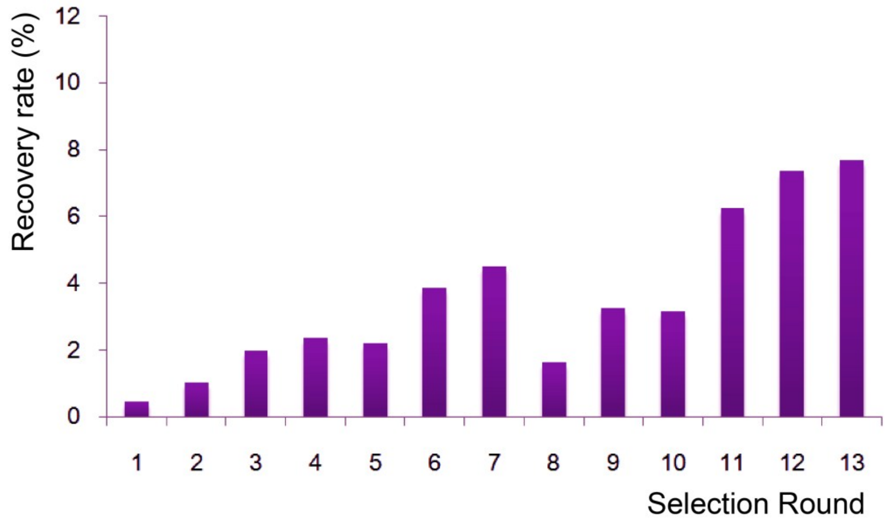

Supplementary Figure 1. Enrichment of ssDNA pool during bacteria-SELEX. The bar graph shows the amount of ssDNA recovered from the supernatant in each selection round.

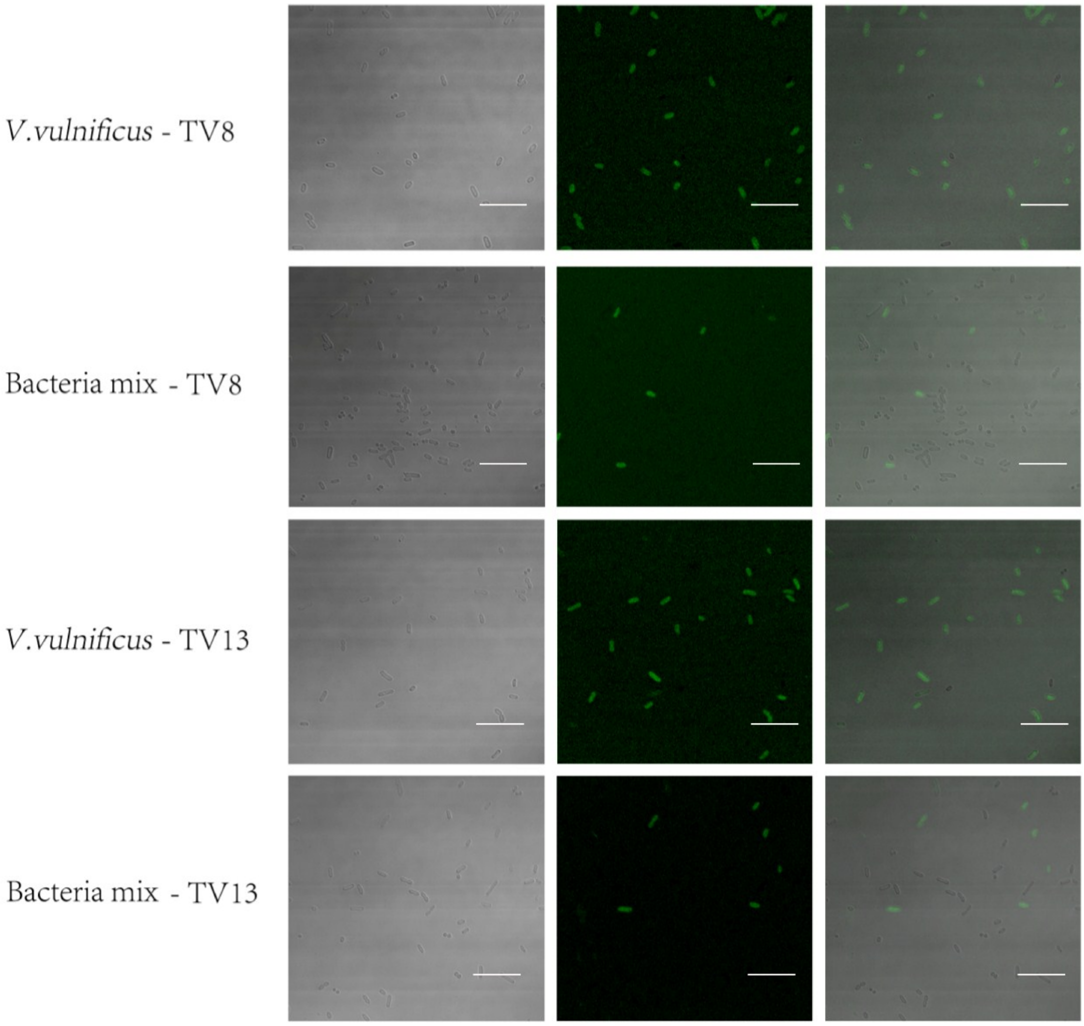

Supplementary Figure 2. Binding ability of TV8, V13 were evaluated by confocal fluorescence microscopic. When incubated together with *S. aureus* and *C. albicans*, TV8 and V13 can successfully pick *V. vulnificus* out. (Scale bar, 10 µm)

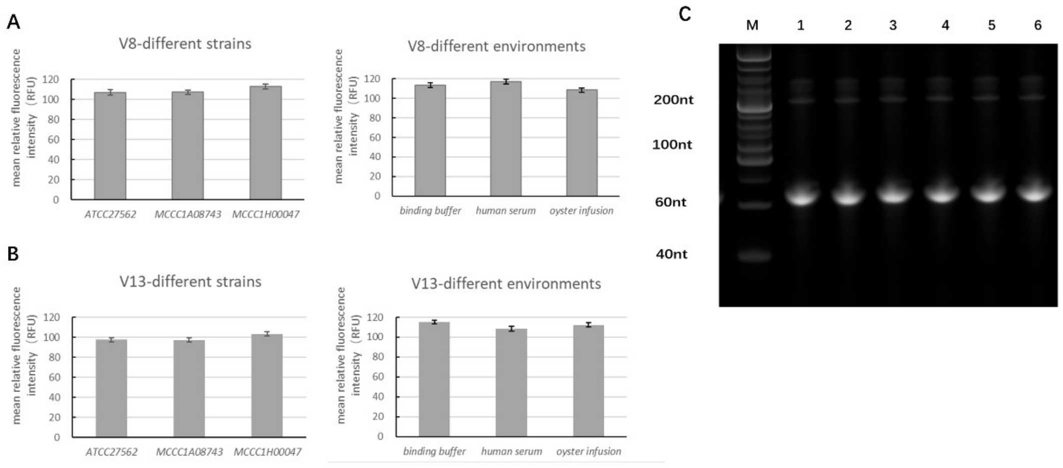

Supplementary Figure 3. Effect of different strains and different binding environment on binding ability of V8 and V13 A. The comparison of binding performance of V8 and V13 on different *V.vulnificus* strains and in different binding environments. **B.** The comparison of binding performance of V8 and V13 on different *V.vulnificus* strains and in different binding environments. **C.** Gel electrophoresis results of aptamers incubated in different environments. Lane 1,V8 in binding buffer. Lane 2, V8 in human serum. Lane 3, V8 in oyster infusion. Lane 4, V13 in binding buffer. Lane 5, V13 in human serum. Lane 6, V13 in oyster infusion.

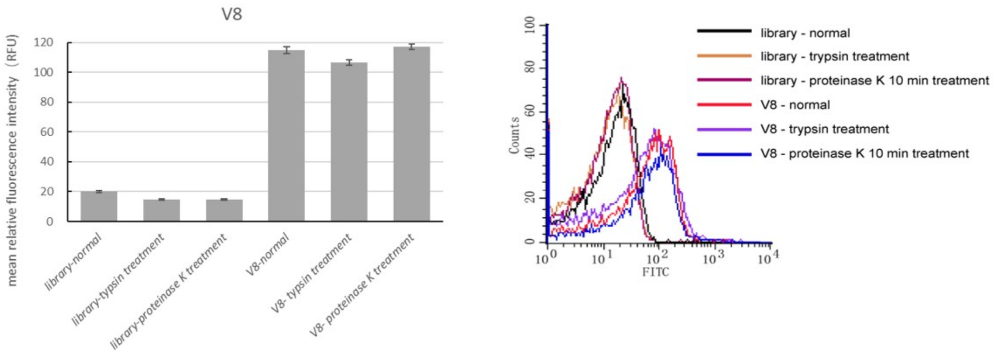

Supplementary Figure 4. Aptamer V8 bind to protein-free *V. vulnificus*

10min of proteinase K treatment and 30 min of trypsin treatment on *V. vulnificus* did not cause significant fluorescent signal change compare to wild *V. vulnificus* cells. Bar plot and flow cytometry results are showed simultaneously.
